# Distinct brain responses to different inhibitions: Evidence from a modified Flanker Task

**DOI:** 10.1038/s41598-017-04907-y

**Published:** 2017-07-27

**Authors:** Liufang Xie, Maofan Ren, Bihua Cao, Fuhong Li

**Affiliations:** School of Psychology, Jiangxi Normal University, Nanchang, 330022 China

## Abstract

Whether inhibition is a unitary or multifaceted construct is still an open question. To clarify the electrophysiological distinction among the different types of inhibition, we used a modified flanker paradigm, in which interference inhibition, rule inhibition, and response inhibition were compared to non-inhibition condition. The results indicated that, compared to the non-inhibition condition (1) the interference inhibition condition induced larger negativities during N2 epoch at the frontal region, (2) the rule inhibition condition elicited a larger N1 at the posterior region, followed by a larger P3a at the frontal region, reflecting the function of proactive cognitive control in the new stimulus-reaction (S-R) association, and (3) the response inhibition condition evoked a larger P3b at the posterior region, reflecting the process of suppressing the old response and reprogramming the new action. These findings provide new evidence that distinct neural mechanisms underlie different types of inhibition.

## Introduction

Inhibition is a core component of executive function^1–6^. Inhibition refers to the processes of suppressing information that is not (or no longer) relevant for the current processing in working memory^7, 8^. Inhibition also refers to suppressing a stimulus that pulls for a competing response so as to carry out a primary response or an appropriate action, suppressing distractors that might slow the primary response, or suppressing internal stimuli that may interfere with the current operations of working memory^4–6, 8–10^.

Whether inhibition is a unitary construct or a multidimensional construct has long been controversial. Recently, several theorists have proposed that inhibition-related processes are a family of functions rather than a single unitary construct^11, 12^. For example, Shiffrin and Schneider^13^ initially proposed controlled inhibition (the conscious and deliberate suppression of irrelevant stimuli or responses) and automatic inhibition (occurs without awareness and appears to be involuntary). Harnishfeger^14^ proposed that inhibition processes could be classified according to the following three dimensions: 1) whether they are intentional or unintentional, 2) whether inhibition takes place at a behavioral or cognitive level, and 3) the distinction between inhibition and resistance to interference. Nigg^11^ listed four types of inhibitory control functions, including interference control, cognitive inhibition, behavioral inhibition, and oculomotor inhibition. Recently, other types of inhibition including response or motor inhibition, lateral inhibition, prepulse inhibition, inhibition of return, and semantic inhibition have also been proposed^6, 15–23^. Moreover, Duncan’s Multiple Demand system theory^24^ and Niendam’s meta-analysis of fMRI data^25^ suggest a common network that underlies diverse inhibitory processes; however, a number of imaging studies found that the activation within the network differs between different types of inhibitory processes^26–29^.

Although inhibition-related functions may be conceptually distinguishable, the neural distinctions between different types of inhibition are not well understood. Recently, a few studies compared the neural mechanism of interference control with response inhibition. For example, Brydges *et al*.^30^ combined a flanker task and go/no-go task and revealed that slightly different brain areas are activated during interference suppression and response inhibition, supporting a neuroanatomical division^12, 31–33^. Groom and Cragg^34^ compared the response inhibition and conflict/flanker suppression in a hybrid go/no-go flanker task. They found that N2 amplitude was larger in incongruent than congruent trials; however, it was not enhanced by response inhibition when the stimulus array was congruent. Instead, P3 amplitude was greater in trials requiring response inhibition^34^.

In the current study, we attempted to test the distinctions among three types of inhibitions using a modified version of flanker task. The three inhibitions were flanker inhibition (i.e., interference control), rule (cognitive) inhibition, and response inhibition. It is necessary to note that, the response inhibition in the current study is different from that in the go/no-go or stop signal task. Response inhibition here refers to the process of implementing one response while inhibiting other alternative responses when there were two or more competitive responses^35^.

The flanker task is a typical task demanding interference control^36–38^. In a letter flanker task, participants have to focus on a central letter and ignore surrounding letters (i.e., the flankers) that could be identical (i.e., congruent) or different (i.e., incongruent) to the central letter. Increased reaction times (RTs) and decreased accuracies are observed for incongruent as compared to congruent stimuli^37^. It has been found that the amplitude of the N2 component, peaking 200–400 ms post-stimulus and typically maximum at the frontocentral scalp locations, is enhanced following incongruent relative to congruent arrays^34, 39–42^. The enhanced N2 have been interpreted as the suppression of irrelevant stimuli^42–45^.

Cognitive/rule inhibition has been widely studied in directed forgetting or set-shifting task^11, 46^, in which the suppression of irrelevant information or invalid rule from working memory is required. For example, in the set-shifting task, the task-set reconfiguration requires the inhibition of the prior task-set^46, 47^. Although psychometric studies suggest that set-shifting may be conceived of as separate executive functions^2, 48^, it is agreed that shifting performance necessarily involves inhibitory control^25, 49–53^. Previous event related potential (ERP) studies on set-shifting have shown that a number of ERP components, including the N1, P2, N2, and P3, are evoked by shifting trials. N1 at parietal sites is associated with attentional selection processes such as focusing on task-relevant stimuli^54–56^, and variation in N1 amplitude reflects a change in demand of transient inhibition by the proactive attention control^57^. The P2 may be sensitive to an early task-set updating process that would rapidly “detect” a relevant change in the task when a shift is involved^58, 59^. The shift-sensitive increase in N2 amplitude has been considered as an index of a process of inhibition of the currently irrelevant task-set^60^ and intentional control of motor responses, which is applied to enable the appropriate response^61^. Moreover, previous studies also revealed P3 attenuation on shifting trials, which is possibly related to higher task complexity compared to repeat trials^59, 61, 62^. P3 amplitude is larger in switch trials than non-switch trials when participants are required to keep more information active in working memory^63–65^.

If the current trial in a flanker task had the identical stimulus and the same response as the preceding trial, there would be a response repetition benefit^66–70^. In the classical choice RT tasks, identical stimulus-reaction (S-R) trials are usually associated with particularly fast responses, which is suggested to reflect remission of the same and the most recent response on detection of an identical stimulus reoccurrence^71–73^. In contrast, when the stimulus is different from the preceding trial, the preceding response might be partially primed before preparing the current response^73–76^. Thus, an inhibition of the preceding response and the subsequent reprogramming of new response would be triggered^35, 77^. It has been shown that P300 amplitudes under the response-changed condition were enhanced as compared to the response-unchanged condition^35^.

The goal of the current study was to elucidate the neural distinction between the above-mentioned three inhibition-related functions using a modified flanker task, in which the rules regarding the S-R association were switched unpredictably. Previous studies had adopted the flanker task in task-switching paradigm to address the performance monitoring, behavior adaptation, conflict adaptation, attentional switching, and task-set inhibition^41, 61, 78–82^. To our knowledge, no studies have explicitly compared these two inhibition functions (rule inhibition and flanker inhibition) and response inhibition in the same task. Particularly, we defined a baseline condition, under which the rule, stimulus, and response in the current trial were identical to the directly preceding trial. Besides, the flankers did not appear in the baseline condition.

Previous studies on flanker control usually considered whether the current trial is a congruent trial or not and did not exclude the influence of the preceding incongruent trial on the current trial. In our opinion, if the preceding trial was an incongruent trial, there would be a conflict-adaptation effect on the current trial, which was demonstrated by numerous studies^83–86^. Because of this consideration and the comparability between experimental conditions, we selected the incongruent trials that preceded with a congruent trial as the flanker trial in the current study. Thus, the flanker condition in present study can be seen as a pure flanker. Previous studies have found that the frontal N2 is more negative in the flanker condition relative to non-flanker^34, 39–42^. Accordingly, the flanker inhibition condition in the present study was also expected to induce a more negative N2 than the baseline.

Compared to the interference (flanker) inhibition, the rule inhibition is one type of executive control accompanied by working memory manipulation because subjects need to suppress the old rule in working memory while turning to and maintaining the new rule, so as to implement the new rule to the new stimuli quickly and accurately. Previous studies found that rule inhibition was mainly reflected during the N2-P3 epoch^60–65^, and particularly the frontal P3a evoked by attention-switching for incoming stimuli^87–91^. Hence, we expected that the rule inhibition trials might evoke a larger P3a than the baseline, reflecting the process of constructing and keeping a new S-R association in the working memory^92^. Moreover, in our study, the rule inhibition processes started earlier from the negative feedback of the preceding trial till the offset of the current trial. The proactive control is presumably involved in this somewhat long period of inhibition, and the central execution system might allocate more attention resources to the new task-set and stimuli. Correspondingly, we expected that, compared with the baseline, the rule inhibition condition might also elicit greater amplitudes in the early ERP components, such as N1 that was associated with attention allocation^54–56^ and P2 that was sensitive to the early rule change^58, 59^.

Previous relevant studies did not dissociate the response inhibition from flanker inhibition, so it is not easy to provide a specific anticipation about the ERPs characteristic of response inhibition in flanker task. Fleming, Mars, Gladwin, and Haggard (2009)^35^ found that response-change and response-unchanged conditions had distinct electrophysiological responses during the P3 component^35^. Accordingly, we expected that, in comparison to the baseline condition, the response inhibition condition would elicit a large P3b at the posterior brain, indexing the process of response inhibition and the reprogramming of a new action.

## Results

### Behavioral Data

Accuracy was calculated for each condition as the mean percentage of the correct responses of all trials. RTs were calculated as the mean RT for correct responses in each condition. RTs and accuracies for each condition (flanker inhibition, rule inhibition, response inhibition, and non-inhibition) are shown in Table 2 . The repeated measures analysis of variance (ANOVA) analysis on RTs and accuracies both showed a significant main effect of condition [*F*_RT_(3,57) = 22.78, *p* < 0.001, *ƞ* ^2^ = 0.55; *F*_accuracy_(3,57) = 21.42, *p* < 0.001, *ƞ* ^2^ = 0.53]. Post-hoc tests revealed that the RTs for flanker inhibition and response inhibition conditions were significantly longer than the RTs in non-inhibition condition respectively (*p*_response_ = 0.022, *p*_*flanker*_ < 0.001). The accuracies for flanker inhibition and rule inhibition conditions were significantly lower than that of non-inhibition condition respectively (both *p*

**Table 1:** Sample stimuli used in different conditions.

Condition	Preceding stimulus	Feedback	Current stimulus
Flanker Inhibition	↓↓↓↓↓↓		↑↑↓↑↑
Rule Inhibition	↑↑↑↑↑		↓↓↓↓↓
Response Inhibition	↑↑↑↑↑		↓↓↓↓↓
Non-inhibition	↑↑↑↑↑		↑↑↑↑↑

< 0.001).

### ERP Data

**Figure 1:**
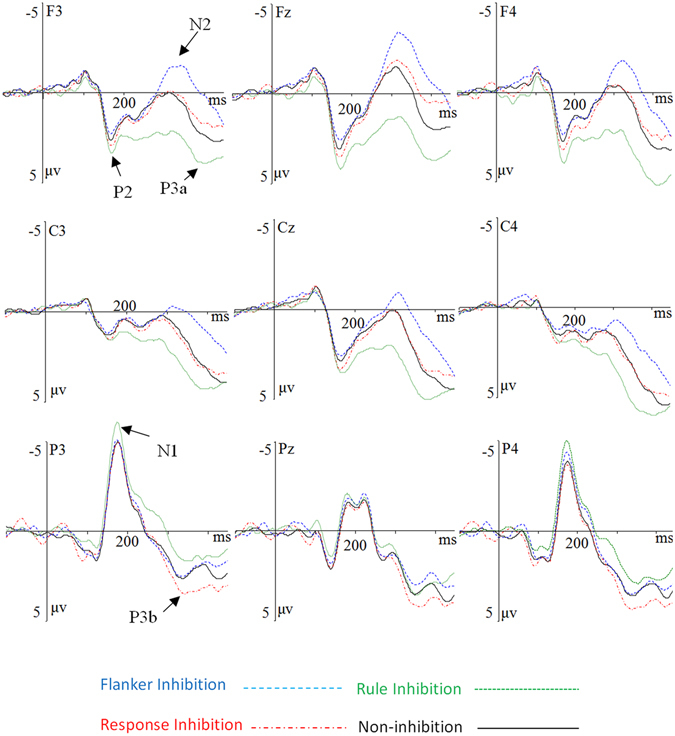
Grand-averaged ERP waveforms. The grand-averaged ERP waveforms are shown for each condition according to nine sites. The zero point on the time axis indicates the stimulus onset.

**Figure 2:**
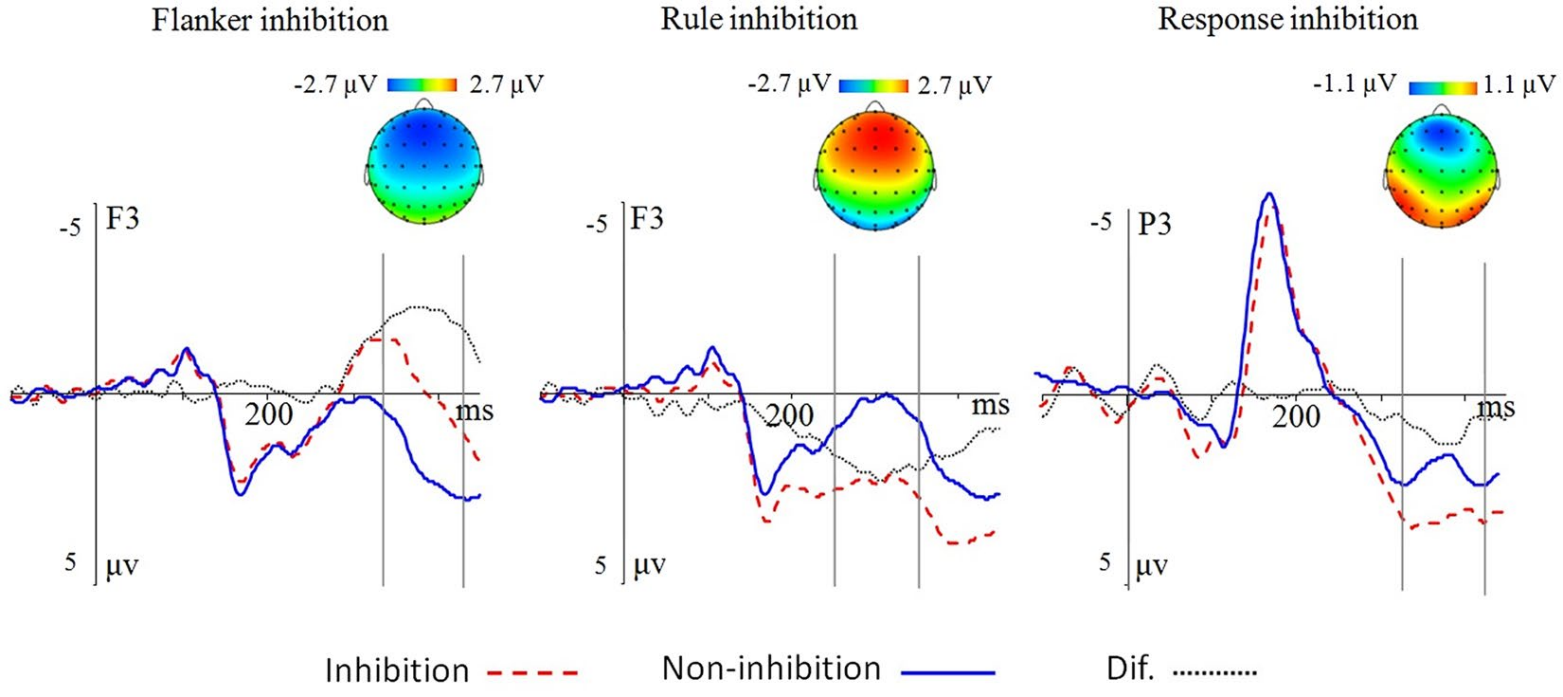
The difference waveforms and topographic map. The topographic map of difference waveforms under the three conditions. The zero point on the time axis indicates the onset of stimuli.

The grand-average ERP waveforms and the different waveforms and topographic maps are shown in Figs 1 and 2. The ANOVA on the posterior N1 amplitude revealed a main effect of condition, *F*(3,57) = 5.12, *p* = 0.010, *η* ^2^ = 0.21. Post-hoc tests revealed that the rule inhibition condition elicited a larger N1 than non-inhibition condition (*p* = 0.038), whereas both the flanker inhibition and response inhibition conditions had no significant differences from the non-inhibition condition respectively (both *p* > 0.05); there was no significant difference between other pairs of conditions (all *p* > 0.05). The electrode also had a main effect, *F*(15,285) = 14.88, *p* < 0.001, *η* ^2^ = 0.44. There was an interaction between condition and electrode, *F*(45,855) = 6.14, *p* < 0.001, *η* ^2^ = 0.24 (Table 3). The simple effect analysis found that, larger N1 amplitudes were evoked by rule inhibition condition as compared with non-inhibition condition at the parietal electrodes including P3, P5, PO3, P4, P6, and PO4 (all *p* < 0.05); and increased N1 was evoked by rule inhibition condition as compared with the response inhibition at the most of right parietal-occipital electrodes including P2, P4, P6, PO4, and PO3 (all *p* < 0.05). ﻿Please see the supplementary Table S1 for the detailed results.﻿

**Table 2:** Mean (standard deviation) reaction time and accuracy for different conditions.

Condition	Flanker Inhibition	Rule Inhibition	Response Inhibition	Non-inhibition
RT (ms)	559 (11)	485 (18)	490 (10)	470 (9)
Accuracy (%)	87.0 (0.02)	87.8 (0.02)	95.3 (0.01)	96.8 (0.01)

The ANOVA on the frontal P2 component showed a main effect of condition, *F*(3,57) = 7.59, *p* = 0.002, *η* ^2^ = 0.29. Post-hoc tests revealed that the rule inhibition condition elicited larger P2 amplitudes than the non-inhibition condition (*p* = 0.010) and flanker inhibition condition (*p* = 0.024), whereas the flanker inhibition and response inhibition conditions had no significant difference from the non-inhibition condition respectively (both *p* > 0.05); there was no significant difference between other pairs of conditions (all *p* > 0.05). The electrode also had a main effect, *F*(15,285) = 7.09, *p* < 0.001, *η* ^2^ = 0.27. There was no interaction between condition and electrode.

For the frontal N2, the results of ANOVA revealed a main effect of condition, *F*(3,57) = 33.99, *p* < 0.001, *η* ^2^ = 0.64. Post-hoc tests revealed that the flanker inhibition condition elicited the largest N2 amplitudes than other conditions (all *p* < 0.001); while the rule inhibition condition elicited the smallest N2 than other conditions (all *p* < 0.001). The response inhibition condition had no significant difference from the non-inhibition condition (*p* > 0.05). The electrode also had a main effect, *F*(15,285) = 8.47, *p* < 0.001, *η* ^2^ = 0.31. There was an interaction between condition and electrode, *F*(45,855) = 6.38, *p* < 0.001, *η* ^2^ = 0.25. The simple effect analysis found that flanker inhibition condition evoked larger N2 amplitudes than the response inhibition and rule inhibition conditions at all of the selected electrodes (all *p* < 0.01), and evoked an increased N2 than the non-inhibition condition at the most of electrodes excluding FC5; the rule inhibition condition had significantly attenuated N2 amplitude than the non-inhibition and the response inhibition condition at most of electrodes excluding FC6 (all *p* < 0.05).

The results of ANOVA revealed a main effect of condition during the P3a component, *F*(3,57) = 38.24, *p* < 0.001, *η* ^2^ = 0.67. Post-hoc tests showed that, the rule inhibition condition elicited a larger P3a than the non-inhibition condition and other two types of inhibitions (all *p* < 0.001), whereas the frontal P3a in the response inhibition condition did not differ significantly from that of the non-inhibition condition (*p* > 0.05). The electrode also had a main effect, *F*(15,285) = 6.94, *p* < 0.001, *η* ^2^ = 0.27. There was an interaction between condition and electrode, *F*(45,855) = 3.57, *p* < 0.001, *η* ^2^ = 0.16. The simple effect analysis did not revealed any suggestive results.

The ANOVA on the parietal P3b component showed a main effect of condition, *F*(3,57) = 4.89, *p* = 0.017, *η* ^2^ = 0.21. Post-hoc tests showed that the response inhibition condition elicited a larger P3b than the non-inhibition (*p* = 0.030) and the flanker inhibition condition (*p* = 0.002), whereas both the rule inhibition and flanker inhibition conditions overlapped with the non-inhibition condition (both *p* > 0.05). The electrode also had a main effect, *F*(15,285) = 6.52, *p* < 0.001, *η* ^2^ = 0.26. There was an interaction between condition and electrode, *F*(45,855) = 9.33, *p* < 0.001, *η* ^2^ = 0.33. The simple effect analysis revealed the marked difference between the response inhibition and non-inhibition conditions at the following sites, P3, P5, P6, and PO4 (all *p* < 0.05); the marked difference between the response inhibition and the flanker inhibition condition at the following sites, P1, P2, P3, Pz (all *p* < 0.05); and the marked difference between the response inhibition and rule inhibition at the P3, P5, PO3, P4, P6, and PO4 (all *p* < 0.05).

## Discussion

In the present study, we recorded the ERPs evoked by different types of inhibitions in a modified Eriksen Flanker task. The RTs for the response inhibition and flanker inhibition trials were significantly longer than that of the non-inhibition trials. The accuracies for the flanker inhibition and rule inhibition conditions were significantly lower than that of the non-inhibition condition. The results of the behavior data indicates a profound increase in RT and a decrease in accuracy under the flanker inhibition condition, which is consistent with the typical finding on the flanker congruency effect^36–38, 93^. Compared to the non-inhibition condition, the rule inhibition trials were less correctly responded to, which was in line with the results of previous studies^46, 94–96^. The RTs of rule inhibition trials had no significant difference from the non-inhibition condition, which is different from the results of the previous studies^46, 78^. One possibility is that there is enough preparation time (at least 1800 ms between switching cue and target stimulus) to reduce the switch cost^39, 62, 96, 97^. Another possibility is that participants received the rule-switch signal via the negative feedback (e.g., red square) to the preceding trial; hence, a proactive control was triggered before the presentation of the current trials^98^. It is likely that these two possible factors cause the non-significant difference in RT between the rule inhibition and non-inhibition conditions. The RTs for the response inhibition condition was significantly longer than the non-inhibition condition, reflecting response inhibition and response change^66, 67, 77^.

We also found different brain responses under different inhibition conditions. The flanker inhibition condition elicited a larger N2 component than the non-inhibition condition at the anterior brain scalp. The rule inhibition elicited a positive-going component during the N2-P3 time window, at the anterior region. Particularly, an increased N1 was observed at the posterior region in the rule inhibition condition. The response inhibition condition evoked a larger posterior P3b than the non-inhibition condition and other two types of inhibitions.

The flanker task in the present study was performed in a rule switch paradigm. Consistent with our expectation, the flanker inhibition condition elicited a more negative N2 than the non-inhibition condition and other two types of inhibitions at the frontal sites. The N2, with a frontocentral topography, is associated with cognitive control^35, 42, 98, 99^, particularly related to the suppression of irrelevant stimuli^40^. Kopp *et al*.^42^ found that the N2 amplitude was larger in incongruent trials than congruent trials^39^. The incongruence effect correlation with N2 component has been replicated by a number of studies^30, 34, 37, 38, 42, 100^. The larger N2 evoked by flanker inhibition in the present study is consistent with the explanation in terms of interference control^17, 28^ and possibly reflects the function of reactive control^101–103^.

Proactive control might contribute to the significant difference between the rule inhibition and non-inhibition conditions at the whole epoch. First, the electrophysiological differences initially emerged at the posterior N1 (150 ms) component. At this time window, the rule inhibition condition elicited a larger N1 than the non-inhibition condition. N1 component found in the anterior and posterior sites differed in latencies and the underlying cognitive functions^55^. The frontal N1, which peaked around 100 ms, is often correlated with response-related processes, and the parietal-occipital N1 peaked around 160 ms, which might reflect the discriminative process and perceptual load^104–106^. N1 also reflects the demands of the recruitment of cognitive control after stimulus presentation^107^. We speculated that proactive control triggered in the rule inhibition condition might evoke increased cognitive control on the processing of the target that followed the rule-switching signal; subsequently, increased N1 amplitudes were evoked in the rule inhibition condition as compared to the non-inhibition condition^56, 57, 107–109^.

Under the rule inhibition condition, there was no flanker in the current and preceding trials. However, the direction of all arrows in the current trial was opposite to that of preceding trial (e.g., from ↑↑↑↑↑ to ↓↓↓↓↓). We found that larger anterior P2 amplitudes were elicited in the rule inhibition condition as compared to the non-inhibition and the flanker inhibition conditions, which might reflect the detection and processing of the global change, such as arrow direction of the stimuli array^54, 110^. Compared to the non-inhibition condition and the other two types of inhibitory processes, the rule inhibition condition induced a larger frontal P3a. Squires, Squires, and Hillyard^87^ first distinguished the P300 into the frontal P3a (300 to 400 ms) and posterior P3b (350–600 ms). The P3a has a central peak and seems to reflect shifts or allocation of perceptual attention, and this process reflects the top-down attention switching for incoming stimuli over response processes to distracters^88–90^. This indicates that the P3a may reflect the activation of a more general brain switching mechanism responsible for processing both stimuli and task novelty, and that an enhanced P3a is elicited by switching of a rule and hence might be an index for the detection of changes in task sets, which may require changes in responses^91^. In the present study, increased P3a observed in the rule inhibition condition might reflect the detection of changes in task requirement and the preparation for action reorganization of the new task-set.

At the posterior site, the response inhibition condition evoked a more positive P3b than the non-inhibition condition and the flanker inhibition condition. P3b has been suggested to be associated with the execution of the task or the process of responding. Donchin^111^ suggested that the posterior P3b reflects working memory context updating, the comparison of the attributes of incoming stimuli with an internal model, and the subsequent revision of the model^63, 64, 88, 90, 91, 112, 113^. P3b amplitude is proportional to the total working memory required during task performance. In comparison to the non-inhibition condition, in which stimulus and response in the current trial were both identical to the preceding trial, the stimulus and response changed from the preceding trial to the current trial in the response inhibition condition. Consequently, participants should inhibit the repetition of the foregoing response, while reprogramming a new action in working memory. Thus, the increased P3b in the response inhibition condition might reflect the inhibition of the old S-R association as well as the reprogramming of the action during response shifting^35^.

In summary, we found the different behavioral and brain responses across different brain regions in different time windows. Rule inhibition begins early in the posterior N1 components, reflecting the function of proactive control. During the N2 time window, the flanker inhibition condition evoked a larger negativity than other conditions at the frontal sites, reflecting a reactive control process. In comparison with non-inhibition condition, the rule inhibition condition induced a larger P3a, while the response inhibition condition evoked a larger P3b during the P3 time window. These finding implies that different types of inhibitions might be controlled by different brain regions, at least with different temporal courses. However, the following limitations should be addressed in future studies. First, the present study compared each type of inhibition with the baseline respectively, and only analyzed the somewhat pure flanker trials. In fact, flanker inhibition stimuli also incorporate response inhibition. Future study might calculate a difference wave between flanker inhibition and response inhibition to remove any aspects of response inhibition that may be included in the flanker inhibition trials. Second, as compared to other two types of inhibitions, rule inhibition condition not only involved the process of inhibiting the old rule but also accompanied with the process of shifting to the new rule; hence, future studies are needed to distinguish the shifting component from the rule inhibition condition. Third, the rule inhibition condition was necessarily triggered by a negative feedback; hence, the method of elucidating the effect of feedback also remains unaddressed.

## Methods

### Participants

After obtaining informed written consent, 20 undergraduate volunteers (8 male subjects, aged from 18 to 21 years, mean (*M*) = 19.35 years, standard deviation (*SD*) = 1.17) participated in this study. All subjects reported to be right-handed, and all had normal or corrected-to-normal eyesight and reported no neurological disorders. All participants were paid for their participation. The study was approved by the Ethics Committee of Jiangxi Normal University (China), and the investigation was carried out in accordance with the latest version of the Declaration of Helsinki.

### Materials and procedures

In a modified Eriksen-Flanker task^76^, five vertical direction arrow-strings consisting of “↑” and “↓” were used as congruent (↑↑↑↑↑, ↓↓↓↓↓) and incongruent stimuli (↓↓↑↓↓, ↑↑↓↑↑). These stimuli were presented in a pseudorandom order with 55% incongruent stimuli of all trials. In terms of the relationship between the current trial and the directly preceding trial, the following four types of trials were defined. In the flanker inhibition trials, the preceding stimulus was a congruent one, whereas the current stimulus was an incongruent trial with the same S-R rule and the same button response to the preceding trial (Table 1). When an incongruent stimulus appeared in the current trial, if the S-R rule or the correct response button was not the same as that in the preceding trial, or if the preceding trial was not a congruent trial, these trials were not defined as flanker inhibition trials. In the rule inhibition trials, the first trial followed the switch signal and was a congruent stimulus with the same button response as the preceding trial. In the response inhibition trials, both the preceding and the current stimuli were congruent trials; however, the corresponding buttons were different. In non-inhibition (baseline) trials, the current stimulus was a congruent trial (non-flanker) and was identical to the preceding trial.

**Table 3:** Statistical (*F* and *P*) values of ANOVA on each components.

Time Window (ms)	Component	Main effect				Interaction effect	
		Condition		Electrode		Condition × electrode	
		*F*	*P*	*F*	*P*	*F*	*P*
150–200	Posterior N1	5.12	0.010	14.88	0.000	6.14	0.000
270–350	Frontal P2	7.59	0.002	7.09	0.000	1.83	0.074
	Frontal N2	33.99	0.000	8.47	0.000	6.38	0.000
350–450	Frontal P3a	38.24	0.000	6.94	0.000	3.57	0.001
	Posterior P3b	4.89	0.017	6.52	0.001	9.33	0.000

**Figure 3:**
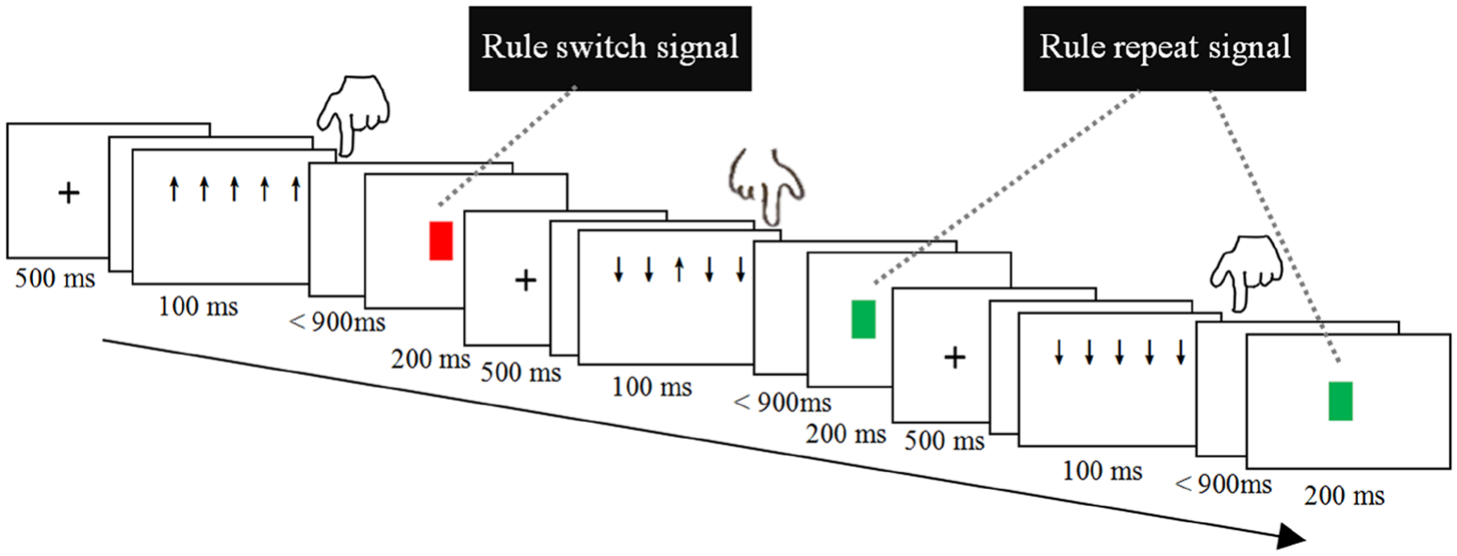
Experiment paradigm and procedure. A participant might respond to the central “↑” by pressing the key “F” with the left hand and received a red square (functioned as a negative feedback, signaling a switch of the task rule).

With each trial, participants were instructed to respond to the central arrow by a button press with either the “F” or the “J” on the keyboard. Participants were not informed about a fixed S-R association. Instead, they had to figure out the currently valid S-R association via feedback, which was presented after each response. The feedback stimulus consisted of a colored square, which was green in case of the correct response and red for an incorrect response. After 6–10 trials, the rule regarding the stimulus–response association was switched. The subjects were not explicitly informed about this switch but had to derive the new rule from the feedback. The first negative feedback (e.g., a red square) functioned as a rule-switch was defined as “switch signal” informing participants to use the opposite S-R rule in the following trials (Fig. 3). Participants were instructed to respond as quickly and accurately as possible and to focus only on the centrally presented arrow (i.e., to ignore the flanker). If the response time exceeded the deadline of 1 s, a feedback comprising a gray square was provided (Fig. 3).

Tasks were presented using E-Prime presentation software (E-Prime 2.0 Professional, Psychology Software Tools, Inc.) with predefined stimuli lists. Stimuli were presented in white against a black background on a screen, viewed at a distance of approximately 60 cm. At the beginning of the task, participants performed training trials. Training was repeated until participants reached an accuracy of at least 60% correct responses. The formal experiment consisted of 1014 trials in total, which were organized into three blocks. Each block lasted about 15 min. The four types of trials (flanker inhibition, rule inhibition, response inhibition, and baseline) were assigned pseudorandomly into the same block, with 80 trials for each type. The same stimuli lists and the experimental procedure were presented to all participants.

A trial started with a cross fixation for 500 ms, followed by a blank screen for a random duration (800–1200 ms). Then, a stimulus was presented for 100 ms and followed by a response screen^41, 61, 78^. After the button press (or exceed 900 ms), a feedback square was displayed for 200 ms, finally, a blank screen (500–800 ms) appeared. The total experiment, including electroencephalogram (EEG) preparation, task training, task run, and breaks, took about 2.5 h.

### Electrophysiological Recording and Analysis

The EEG was recorded using Brain Amp equipment (Brain Products, Germany) with 64 Ag/AgCl ring electrodes, which were mounted on a fabric cap according to the extended 10–20 system. The online reference electrode was placed over FCz, and the ground electrode was placed over AFz. An electrode placed under the right eye (EOG) allowed the monitoring of blinks and vertical eye movements. The impedance at each electrode was kept below 10 kΩ. Raw data were band-pass filtered between 0.01–250 Hz and digitized at a sampling rate of 500 Hz. The offline preprocessing of the EEG signal was performed in Brain Vision Analyzer 2.1 (Brain Products Gmb H). Blinks, ocular movements, and muscle artifacts were detected and removed using an independent component analysis (ICA), implemented in the Brain Vision Analyzer software. Next, extracted epochs (from −100 to 450 ms) of the correct trials were time-locked with the stimuli. The resulting data were baseline-corrected using windows from −100 to 0 ms. In addition, trials containing further artifacts were removed using an automatic procedure. The automatic detection criteria included an absolute difference between two sampling points exceeding 30 μV/ms, peak-to-peak deflections in a segment exceeding ±80 μV within intervals of 200 ms, amplitudes exceeding a value of ±80 μV, and activity lower than 0.1 μV within intervals of 200 ms. Then, data were re-referenced to the average of the two mastoid channels (TP9 and TP10), which are typically used as references in the ERP literature on task-switching. The averaging procedure was performed on the extracted epoch. The single-subject ERP averages for each of the four conditions were used for further analysis. The two mastoid channels were excluded from the analysis. In the final data set, at least 67 artifact-free trials per task condition were contained.

The stimulus-locked ERPs were measured at the following 32 electrodes: AF3, F1, F3, F5, FC1, FC3, FC5, AF4, F2, F4, F6, FC2, FC4, FC6, Fz, FCz, CP1, CP3, CP5, P1, P3, P5, PO3, CP2, CP4, CP6, P2, P4, P6, PO4, CPz, and Pz. Based on the relevant literature^34, 36, 63, 64, 104^ and a visual inspection of the grand-averages, the following ERP components were selected. The frontal P2 and posterior N1 were measured over the time window ranging from 150–200 ms after stimuli onset. The frontal N2 was measured during the 270–350-ms time window. The frontal P3a and posterior P3b both measured at 300–450 ms time window. No obvious peaks were found for some conditions in some time windows (e.g., in the P3a time window, only rule inhibition had a distinct peak), and the visual inspection showed that the latency difference was not obvious; hence, we did not analyze the latency of ERPs. The mean amplitudes of each component were all analyzed using two-factor ANOVA. The mean amplitudes of the frontal P2, N2, and P3a were subjected to a 4 (condition) × 16 (electrode: AF3, F1, F3, F5, FC1, FC3, FC5, AF4, F2, F4, F6, FC2, FC4, FC6, Fz, and FCz) repeated measures ANOVA, respectively. The mean amplitudes of the posterior N1, P3b were submitted to a 4 (condition) × 16 (electrode: CP1, CP3, CP5, P1, P3, P5, PO3, CP2, CP4, CP6, P2, P4, P6, PO4, CPz, and Pz) repeated measures ANOVA, respectively. Greenhouse-Geisser corrections were performed on the *p* values where necessary. Bonferroni correction was used for the multiple comparison.

## Electronic supplementary material

Supplementary Information
