# Supplementary material for: Distinct brain responses to different inhibitions: Evidence from a modified Flanker Task
Source: Sci Rep. 2017 Jul 27;7:6657. doi: 10.1038/s41598-017-04907-y (PMC5532368; doi:10.1038/s41598-017-04907-y)
Supplement: Supplementary file 1 — Supplementary Information [file 41598_2017_4907_MOESM1_ESM.pdf]

Distinct brain responses to different inhibitions: Evidence from a modified Flanker Task

Liufang Xie, Maofan Ren, Bihua Cao, Fuhong Li *

School of Psychology, Jiangxi Normal University, Nanchang, 330022, China

*Corresponding author:

Fuhong Li, E-mail address: **lifuhong@jxnu.edu.cn** (F. Li)

Tel: +86-0791-88121252

School of Psychology, Jiangxi Normal University, Nanchang, 330022, China

Supplementary information

Table S1 -Values of the averaged amplitude of each component in different conditions

Window (ms)	Component	Flanker Inhibition (μv)	Rule Inhibition (μv)	Response Inhibition (μv)	Non-inhibition (μv)
150-200	Posterior N1	-2.27	-2.63	-1.96	-2.05
	Frontal P2	1.92	3.00	2.51	2.11
270-350	Frontal N2	-0.94	2.31	0.09	0.18
350-450	Frontal P3a	0.60	4.05	1.86	2.47
	Posterior P3b	2.45	2.57	3.62	3.00
